# Objective Indicators of Physical Activity and Sedentary Time and Associations with Subjective Well-Being in Adults Aged 70 and Over

**DOI:** 10.3390/ijerph110100643

**Published:** 2014-01-02

**Authors:** Janet Withall, Afroditi Stathi, Mark Davis, Jo Coulson, Janice L. Thompson, Kenneth R. Fox

**Affiliations:** 1 Department for Health, University of Bath, Claverton Down, Bath BA2 7AY, UK; E-Mail: a.stathi@bath.ac.uk; 2 Centre for Exercise, Nutrition and Health Sciences, School for Policy Studies, University of Bristol, Bristol BS8 1TZ, UK; E-Mails: mark.davis.ac.uk@gmail.com (M.D.); jo.coulson@bristol.ac.uk (J.C.); K.R.Fox@bristol.ac.uk (K.R.F.); 3 School of Sport, Exercise and Rehabilitation Sciences, University of Birmingham, Edgbaston, Birmingham B15 2TT, UK; E-Mail: j.thompson.1@bham.ac.uk

**Keywords:** well-being, physical activity, sedentary time, lower limb function, accelerometer, life satisfaction

## Abstract

This study explored the associations of the volume and intensity of physical activity and the volume of sedentary time with subjective well-being in a diverse group of 228 older adults in the UK (111 female, mean age 78.2 years (SD 5.8)). Physical activity (PA) and sedentary behaviour were assessed by accelerometry deriving mean steps per day, mean moderate/vigorous PA minutes per hour (MVPA min·h^−1^) and minutes of sedentary time per hour (ST min·h^−1^). Lower limb function was assessed by the Short Physical Performance Battery. Subjective well-being was assessed using the SF-12 health status scale, the Ageing Well Profile and the Satisfaction with Life Scale. Linear regressions were used to investigate associations between the independent variables which included physical activity (steps and MVPA), sedentary time, participant characteristics (gender, age, BMI, education, number of medical conditions), and lower limb function and dependent variables which included mental and physical well-being. Steps, MVPA and lower limb function were independently and moderately positively associated with perceived physical well-being but relationships with mental well-being variables were weak. No significant associations between sedentary behaviours and well-being were observed. The association between objectively evaluated physical activity and function and subjective evaluations of physical well-being suggest that improving perceptions of physical health and function may provide an important target for physical activity programmes. This in turn may drive further activity participation.

## 1. Introduction

In 20 years time, nearly a quarter of the population in the UK will be aged 65 and over [1]. Not only is the number of older people increasing, but people are living longer lives. Between 1960 and 2010 the average life span increased by around 10 years for a man and 8 years for a woman, with UK life expectancy estimates being 85.6 years for women, and 83 years for men who are now 65 years [2]. Supporting people to live independently and in good health for as long as possible has become a key public health challenge. Physical activity has consistently been shown to improve older people’s physical and mental health [3,4] and to have an important role in the maintenance of independent living [5,6]. Physical activity has been shown to improve aspects of mental well-being such as self-perceptions and mood, and to reduce anxiety and stress in older adults [3,4,7]. Despite this wealth of evidence only a small proportion of older people accumulate 30 min of moderate intensity physical activity (MVPA) on five or more days of the week [8], with only 30% reporting more than ten minutes of MVPA in the previous month [9,10].

In addition to being the least active sector of society, older adults are the most sedentary [11]. Sedentary behavior is defined as energy expenditure of less than 1.5 metabolic equivalents (METs), and is mainly represented by sitting, reclining, or lying down during waking hours [12]. The National Health and Nutrition Examination Survey (NHANES) reported that US adults aged 60–69 were sedentary for an average of 8.5 hours each day [13]. The Health Survey for England (HSE) showed that 50% of older adults aged 65–74 and 65% of those aged 75 and above were sedentary for six or more hours on weekdays [14]. In a UK cohort of older adults (mean age = 78 years) the average sedentary time was over 11 hours each day [15]. With the development of more sophisticated physical activity assessment tools such as accelerometry, it has become clear that sedentariness and low levels of physical activity have separate determinants and independent effects on health and mortality [16,17]. Restricting sedentary time is now recommended in the physical activity guidelines published by the four Chief Medical Officers in UK [8], although there is no specific guidance on the exact amounts of sedentary time that are detrimental to health due to the limited evidence base. 

The research base on the association of sedentary time with subjective well-being, particularly amongst older adults, is limited. Subjective well-being has been defined as a multi-dimensional, phenomenological expression by the individual of the quality of her or his state of existence [9]. In one recent study among a group of kidney cancer survivors, those under 60 years showed a negative association of sitting time with physical and functional aspects of Quality of Life (QoL) but amongst the over 60s there was a surprising positive association between sitting time on a week day and emotional well-being [18]. In another study of men over 55 years no association was found between Health Related Quality of Life (HRQoL) and sitting time on weekdays, although at weekends sitting time was negatively associated with HRQoL when comparing the lowest and highest quartiles [19].

A contributor to the equivocality in the limited evidence base is the reliance on self-report of physical activity and sedentary time. This method is susceptible to poor memory, subjectivity, and socially desirable responding in older adults [20,21,22]. Objective measures offer greater precision and also allow activity at different intensities to be assessed, along with numbers of steps walked. Currently we have limited knowledge of whether the volume or the intensity of activity is more important in influencing well-being and we know little about the relationship between sedentariness and well-being, particularly in those well into older age. This paper reports the use of accelerometry in exploring the relationships between physical activity volume and intensity, volume of sedentary time and subjective well-being in a diverse group of older adults in the UK. 

## 2. Experimental Section

### 2.1. Recruitment

Participants were volunteers for the Older People and Active Living (OPAL) Project, a two year observational study funded by the National Prevention Research Initiative (Phase 1). Project OPAL was designed to provide comprehensive assessment of patterns and levels of activity, functionality, well-being and perceptions of the environment among community dwelling older adults in Bristol, UK. Participants were over 70 years and so likely to be in retirement rather than in transition from employment. Twelve general medical practices within Bristol were selected to provide a 3 × 2 cell recruitment matrix representing localities of low, medium and high index of multiple deprivation (IMD) and high or low proximity to amenities (defined as the nearest store) of their patient catchment area. IMD is an indicator used to characterise the deprivation of Lower Level Super Output Areas in the UK based on several factors including income, employment, health, education, housing, environment, and crime [23]. The overall recruitment rate from those invited to take part was 20.8%, a rate similar to other studies recruiting via general practices [24]. For a full report of recruitment methods see Davis et al. and Fox et al*.* [15,25]. 

### 2.2. Participants

Participants were randomly selected from patient lists in each practice. Those meeting the inclusion criteria, as verified by their General Practitioners (GPs), were mailed an invitation letter, information pamphlet and consent form by the practice administrator. Emphasis was placed on inclusivity in order to attract a full range of levels of health, physical and cognitive function and physical activity. The exclusion criteria included: (1) bereavement within the last two months; (2) terminal illness; (3) suffering from moderate to advanced dementia or other debilitating mental illness; (4) unable to complete a questionnaire with assistance; (5) suffering from an illness that would put the patient at risk by participating; (6) other reasons seen by the GP as relevant. Participants were not significantly different in age and gender to their practice population, and were similar to national samples in terms of BMI scores and level of deprivation of their residence. The study was approved by the Bristol Southmead National Health Service Research Ethics Committee (06/Q2002/127).

### 2.3. Procedures and Measures

Data were collected by trained research assistants over two home visits lasting on average 90 min. Sedentary living and physical activity was assessed by 7-day accelerometry (Actigraph GT1Ms, Pensacola, FL, USA) using a 10-second epoch. At Visit 1, participants were trained to wear the Actigraph on the hip for all waking hours for seven days (with the exception of entry to water). Participants recorded any time spent swimming, but this was not included in the analysis of the accelerometer data. Participants were also asked to complete a daily log recording details of when the Actigraph was worn or removed. In addition, participants were asked to complete a purposes of daily journeys log each day which is described in greater detail elsewhere [26]. Height and weight were measured using portable scales and stadiometer, respectively. Lower limb function was measured by the Short Physical Performance Battery [27] which assesses balance, leg strength and walking speed.

Demographic data, health history (including current health conditions being treated) and well-being variables were collected as part of an interviewer administered questionnaire during Visits 1 and 2. Health related quality of life was assessed using the SF12, a short but reliable measure of perceptions of health status, that provides separate subscale scores for perceived physical (SF-12 Physical) and mental (SF-12 Mental) health [28]. Life Satisfaction, a global statement representing the degree to which the participant feels that life is going well for them, was assessed using the Satisfaction with Life Scale [29]. The Ageing Well Profile (AWP) [30] is a multi-scale measure of subjective well-being providing estimates of social (AWP Social), physical (AWP Physical), mental (AWP Mental) and developmental (AWP Developmental) well-being.

### 2.4. Data Reduction and Analyses

Data from each Actigraph were downloaded using the Actilife Lifestyle Monitoring System version 3.1.3 software. Cases providing a minimum of 10 h of registered wear time for at least five days were included in the analyses. Bouts of more than 100 min of continuous zero count data were considered non wear time and excluded [31]. Data were reintegrated to form one-minute epoch data and were then reduced using Kinesoft (version 3.3.62; Kinesoft Software, Saskatchewan, Canada). The following daily summary variables for each case were computed: mean minutes of moderate to vigorous physical activity (MVPA) (>1,951 counts per min equivalent to >3 METs) per day, mean steps per day (STEPS) [32], and mean number of minutes of sedentary time (0–99 CPM) per day [31]. The count range for sedentary time captured seated activities such as watching TV although standing activities which include no movement might have also been included [31]. Minutes of MVPA were not normally distributed and were subsequently log transformed. 

A bivariate correlation matrix was used to provide unadjusted associations between total volume of sedentary time (min/day), total volume of daily activity (steps/day), physical activity volume (min/day) at moderate-to-vigorous intensity (MVPA), number of medical conditions, lower limb function and aspects of well-being (SF-12 subscales, AWP subscales and Satisfaction with Life scale) to determine salient variables for further analyses.

Regression models were developed for each activity variable (steps, MVPA, and sedentary time) as an independent factor for assessing associations with each of the well-being variables. Prior to the regression analysis multi-collinearity tests were conducted and the results fell within the acceptable range. Model 1 featured the simple relationship between the activity and well-being variables. Model 2 also included participant characteristics (age, gender, education, and BMI) and number of medical conditions. In Model 3 lower limb function was also added. Explained variance in the dependent variable was calculated for each model. 

## 3. Results and Discussion

### 3.1. Participants

Of the 240 participants recruited, 228 provided valid accelerometry data and valid questionnaire data for at least one of the dependent variables. Participants ranged in age from 70–96 years (mean = 78 years) and their characteristics are presented in Table 1. Participants registered a mean of 14.16 ± 1.52 hours of accelerometer data per day. On average, each day participants spent 11.1 hours being sedentary, 18.6 min in MVPA and recorded 4,457 steps. 

**Table 1:** Demographic, MVPA, steps, lower limb function, sedentary time and well-being characteristics of study participants (*n* = 217).

Characteristic	Mean (SD) or N (%)
Men	*n* = 117 (51.35%)
Age (years)	78.2 (5.8)
BMI (kg·m^2^)	27.3 (4.9)
Education level (% tertiary educated)	51.8%
Number of medical conditions	1.9 (1.4)
Minutes of MVPA ^a^/day *	18.6 (20.2)
Steps per day *	4,457.5 (2,483.7)
Sum of scores of lower limb function tests (0–12) ^b^	9.7 (2.4)
Sedentary time per day (hours) ^*^	11.1 (1.6)
SF-12 Physical	21.6 (4.7)
SF-12 Mental	16.0 (2.3)
AWP ^c^ Developmental (5–35)	25.1 (6.1)
AWP ^c^ Physical (5–35)	20.8 (6.1)
AWP ^c^ Mental (5–35)	29.7 (5.3)
AWP ^c^ Social (5–35)	26.5 (6.6)
Sum LS1-5 ^d^ (5–35)	26.4 (5.4)

^a^ MVPA: moderate to vigorous physical activity; ^b^ SPPB: Short Physical Performance Battery; ^c^ AWP: Ageing Well Profile; ^d^ LS: Life Satisfaction; * accelerometer-derived variables are adjusted for minutes of wear time per day.

### 3.2. Physical Activity, Sedentary Time and Markers of Subjective Well-Being

Table 2 shows the zero-order correlation matrix for participant characteristics and activity variables with well-being variables. Weak but significant relationships (*p* < 0.01) were observed, indicating that with greater age and BMI, physical well-being decreases. A moderately strong negative correlation was observed between number of existing medical conditions and the SF-12 Physical, AWP Physical and SF-12 Mental. This is also reflected in a weaker but negative relationship with Life Satisfaction. Lower limb function showed a similar pattern of association. Volume of activity (steps) and moderate to vigorous intensity activity (MVPA) show an even stronger relationship with physical and to a lesser extent with mental aspects of well-being. However, no significant relationships emerged between volume of sedentary time and the well-being variables. 

**Table 2:** Associations of subjective well-being with participant characteristics, steps, MVPA, sedentary time and lower limb function.

	SF-12 Physical	SF-12 Mental	Life Satisfaction	AWP Develop-Mental	AWP Physical	AWP Mental	AWP Social
Gender ^a^	0.179 **	0.186 **	0.154 *	0.087	0.180 **	0.098	−0.048 *
Age (years)	−0.210 **	0.029	−0.089	−0.271 **	−0.207 **	−0.107	−0.239 *
BMI (kg·m^2^)	−0.215 **	−0.068	0.029	0.076	−0.136 *	−0.039	0.085
Highest education level	0.120	0.022	0.107	–0.005	0.152 *	0.057	0.235 *
No of medical conditions	−0.470 **	−0.322 **	−0.177 *	−0.157 *	−0.360 **	−0.186 **	−0.245 *
Steps per day *	0.496 **	0.158 *	0.154 *	0.305 **	0.448 **	0.169 *	0.164 *
Mins of MVPA^b^/d *	0.550 **	0.154 *	0.123	0.288 **	0.454 **	0.140 *	0.307 **
Mins of sedentary time *	−0.102	0.070	−0.012	−0.113	−0.042	0.013	−0.070
Lower limb function^c^	0.545 **	0.242 **	0.212 **	0.368 **	0.474 **	0.198 **	0.343 **

^a^ gender: 0 = female, 1 = male; ^b^ MVPA, moderate to vigorous physical activity; ^c^ Measured by SPPB, Short Physical Performance Battery; * accelerometer-derived variables are adjusted for wear time.

The results of linear regression analyses with SF-12 Physical as the dependent variable are presented in Table 3. Steps per day accounted for 26.1% of the variance and this was increased to 36.3% when gender, age, BMI, education and number of medical conditions were added in Model 2. Lower limb function further increased this to 42.4%. MVPA accounted for 30.1% of the variance and this was increased to 39.3% in Model 2. Lower limb function in Model 3 further increased this to 44.3%. In contrast, volume of sedentary time explained only 1.4% of variance with participant characteristics, with lower limb function increasing the explained variance to 26.2% and 40.8%, respectively. 

The results of linear regression analyses with SF-12 Mental as the dependent variable are presented in Table 4. Neither of the activity or the sedentary variables reached significance in univariate analysis in Model 1. 

**Table 3:** Linear regression analysis on SF Physical scores with participant characteristics, number of medical conditions, lower limb function, MVPA, steps and sedentary time.

	Model 1 ^a^				Model 2 ^b^				Model 3 ^c^			
	B	95% CI	95% CI	P	B	95% CI	95% CI	P	B	95% CI	95% CI	P
Steps/d *	0.001	0.001	0.001	0.000	0.001	0.000	0.001	0.000	0.001	0.000	0.001	0.000
Gender (ref.: female) Male					−0.038	−1.101	1.025	0.943	−0.243	−1.257	0.771	0.637
Age (years)					−0.043	−0.153	0.067	0.439	0.030	−0.079	0.139	0.586
BMI					−0.064	−0.185	0.056	0.293	−0.046	−0.160	0.069	0.432
Education level (1−7)					−0.227	−0.602	0.148	0.233	−0.291	−0.648	0.066	0.109
No. Med. Conditions (1−7)					−1.207	−1.632	−0.782	0.000	−1.173	−1.577	−0.769	0.000
Lower limb function (0−12)									0.668	0.385	0.950	0.000
Model R2		0.261				0.363				0.424		
MVPA/d *	5.761	4.560	6.963	0.000	4.812	3.405	6.219	0.000	2.885	1.274	4.496	0.001
Gender (ref.: female) Male					−0.289	−1.333	0.755	0.586	−0.375	−1.376	0.626	0.461
Age (years)					0.001	−0.104	0.106	0.987	0.037	−0.065	0.139	0.473
BMI					−0.070	−0.180	0.041	0.215	−0.061	−0.167	0.046	0.262
Education level (1−7)					−0.198	−0.561	0.164	0.282	−0.253	−0.602	0.095	0.153
No. Med. Conditions (1−7)					−1.129	−1.534	−0.724	0.000	−1.114	−1.502	−0.725	0.000
Lower limb function (0−12)									0.625	0.339	0.911	0.000
Model R2		0.301				0.393				0.443		
Reg. sed. time/d *	−0.006	−0.013	0.000	0.052	−0.004	−0.010	0.002	0.149	0.000	−0.006	0.005	0.882
Gender (ref.: female) Male					0.408	−0.745	1.561	0.486	−0.165	−1.211	0.881	0.756
Age (years)					−0.145	−0.251	−0.038	0.008	−0.015	−0.117	0.087	0.777
BMI					−0.151	−0.271	−0.032	0.013	−0.095	−0.203	0.013	0.086
Education level (1−7)					0.031	−0.367	0.428	0.879	−0.193	−0.554	0.169	0.295
No. Med. Conditions (1−7)					−1.407	−1.844	−0.969	0.000	−1.226	−1.621	−0.831	0.000
Lower limb function (0−12)									0.902	0.650	1.154	0.000
Model R2		0.014				0.262				0.408		

^a^ Model 1, adjusted for gender, age, BMI, education; ^b^ Model 2, adjusted for (as model 1 +) number of medical conditions; ^c^ Model 3, adjusted for (as model 2 +) lower limb function score; * accelerometer-derived variables are adjusted for wear time.

**Table 4:** Linear regression analysis on SF Mental scores with participant characteristics, number of medical conditions, lower limb function, MVPA, steps and sedentary time.

	Model 1 ^a^				Model 2 ^b^				Model 3 ^c^			
	B	95 CI	95 CI	P	B	95 CI	95 CI	P	B	95 CI	95 CI	P
Steps/d *	0.000	0.000	0.000	0.058	0.000	0.000	0.000	0.273	0.000	0.000	0.000	0.711
Gender (ref: female) Male					−0.141	−0.274	−0.007	0.039	−0.131	−0.264	0.002	0.054
Age (years)					−0.008	−0.022	0.005	0.231	−0.012	−0.026	0.003	0.105
BMI					−0.004	−0.019	0.011	0.605	−0.005	−0.020	0.010	0.524
Education level (1−7)					0.010	−0.037	0.058	0.664	0.013	−0.034	0.061	0.581
No. Med. Conditions (1−7)					0.106	0.053	0.160	0.000	0.105	0.051	0.158	0.000
Lower limb function (0−12)									−0.031	−0.068	0.006	0.104
Model R2		0.013				0.092				0.100		
MVPA/d *	−140	−0.291	0.011	0.069	−0.074	−0.254	0.106	0.420	0.067	−0.145	0.280	0.532
Gender (ref: female) Male					−0.132	−0.265	0.002	0.053	−0.126	−0.258	0.006	0.061
Age (years)					−0.010	−0.024	0.003	0.127	−0.013	−0.027	0.000	0.055
BMI					−0.005	−0.019	0.009	0.491	−0.006	−0.020	0.008	0.419
Education level (1−7)					0.010	−0.037	0.057	0.681	0.014	−0.032	0.060	0.557
No. Med. Conditions (1−7)					0.117	0.064	0.169	0.000	0.115	0.064	0.167	0.000
Lower limb function (0−12)									−0.046	−0.084	−0.008	0.017
Model R2		0.011				0.108				0.129		
Reg. sed. time/d *	0.000	−0.001	0.000	0.472	0.000	−0.001	0.001	0.744	0.000	−0.001	0.000	0.399
Gender (ref: female) Male					−0.137	−0.271	−0.003	0.045	−0.110	−0.243	0.024	0.107
Age (years)					−0.007	−0.020	0.005	0.241	−0.014	−0.027	0.000	0.042
BMI					−0.003	−0.017	0.011	0.650	−0.006	−0.020	0.008	0.401
Education level (1−7)					0.008	−0.039	0.055	0.735	0.018	−0.029	0.065	0.448
No. Med. Conditions (1−7)					0.121	0.070	0.172	0.000	0.112	0.062	0.163	0.000
Lower limb function (0−12)									−0.042	−0.075	−0.010	0.010
Model R2			−0.002			0.105				0.130		

^a^ Model 1, adjusted for gender, age, BMI, education; ^b^ Model 2, adjusted for (as model 1 +) number of medical conditions; ^c^ Model 3, adjusted for (as model 2 +) lower limb function score; * accelerometer-derived variables are adjusted for wear time.

Even after the addition of participant characteristics the explained variance was 10%, rising to 13% when lower limb function was added. Analyses with AWP mental as the dependent variable showed even weaker results with only 2.3–2.7% of variance explained by the activity or sedentary variables.

The results of linear regression analyses with Life Satisfaction as the dependent variable showed a significant relationship only with steps per day, accounting for 2.1% of the variance. MVPA and sedentary time did not reach significance. With the addition of participant characteristics and lower limb function, the total explained variance in Life Satisfaction for any of these regressions was less than 4%. Analyses for the AWP Social scale showed weak results with 2.2% or less of variance explained. Steps per day explained 7.7% of the variance in AWP developmental; however the initial significance disappeared when other factors were added.

This study provides insight into the associations between physical activity and sedentary time and aspects of subjective well-being in 228 community-dwelling older adults. The unique contribution is that activity, sedentary time and lower limb function were assessed using well-established objective methods. The literature is sparse with this particular age group whose average age was 78 years.

The prediction of perceptions of physical well-being demonstrated the strongest and most consistent relationships. Physical well-being is largely a self-summation of how well the individual perceives themself to be able to function physically and be in good physical health. Both steps per day (representing volume of activity) and MVPA (intensity of activity) explained a high percentage of variance. In both cases numbers of existing medical conditions and lower limb function explained even more of variance. Clearly being active, being free from disease or disability, and retaining a high level of physical function are all very important for individuals to feel good about their physical selves and contribute to physical well-being. These findings provide support for the link between physical activity and perceptions of physical well-being [30,33]. 

Conversely, physical activity (either steps or MVPA) showed weak relationships with the psychological variables (SF-12 Mental, Life Satisfaction, AWP mental or AWP developmental). This finding is in contrast with much of the literature relating to older adults [8,34]. However, few of these studies use objective measures of physical activity, with most assessing physical activity through self-report. Fox et al*.*, [35] also found weak relationships between objectively measured physical activity and well-being. Several explanations for this low level of correspondence between activity and psychological aspects of well-being can be suggested. First, qualitative and quantitative data [36,37] indicate that few older people undertake physical activity as a leisure pursuit. Physical activity is seen by people of this age as utilitarian. It functions as a means to achieving their other necessary and desired goals in life. This is supported by data [26] indicating that physical activity primarily arises through completion of daily tasks such as shopping and visiting friends. As long as people maintain the ability to perform these tasks then their psychological well-being might not be influenced by levels of physical activity. However, a decline in physical function leading to lower physical activity and a subsequent lower functional ability leading to loss of independence will influence subjective well-being evaluations. In this study, participants’ physical function might have been sufficiently high to allow them to enjoy good levels of well-being. However, the importance of physical activity for maintenance of physical function [38] stresses the need to identify effective ways of promoting physical activity among older adults. Promotion of exercise as leisure time physical activity might not be the most effective strategy as for many older adults, exercise is associated with negative connotations and is something that has to be endured rather than enjoyed. It is also possible that as people get older, they undertake a process of selection, optimization, and compensation in relation to their daily activities [39,40]. Feeling unable to continue all their activities they select the most rewarding and let the others go. They do fewer things but do them well and so think highly of what they do and their achievements. Therefore, throughout the years they might have adjusted well to a less active lifestyle which still allows them good levels of well-being through fewer but highly valued activities.

The research base into sedentary time and well-being is extremely limited [41]. This study found no associations between sedentary time and measures of well-being, whereas some recently published cross-sectional studies conducted with overweight minority women (18+ years) [42], Latino adults [43] (80% of sample <60 years), a multi-ethnic Asian population in Singapore (Mean Age 43 years) [44] and patients being treated with methadone [45] (Mean Age 39.9 years) have reported some positive associations between high levels of sedentary time and lower levels of well-being. Other cross-sectional studies have shown associations to have been moderated by age [18]; however in a cohort of 375 older men no relationship was found between health related quality of life and sitting time on weekdays [19]. None of these studies used objective measures of physical activity or sedentary time. 

These age-related differences in perceptions of well-being and its relation to sedentary time identified in the somewhat limited literature base may indicate that sedentary time is regarded differently by older adults compared to other populations. High levels of sedentary time, which reflect poor health or a lack of employment in a younger population, may be regarded more positively as a well-deserved chance to rest and relax by older adults. Even in active older people, we observe high levels of sedentary time [15]. The impact of social norms (it is normal to be sedentary when you are older) and the long-established habit of being sedentary might also explain the lack of a negative impact of sedentary time on measures of well-being. 

These findings confirm the importance of retaining good physical function and avoiding debilitating diseases and conditions. The Chief Medical Officers’ report [8] makes it clear that this can best be achieved through regular physical activity. Identifying effective lower limb function programmes that can be accepted and adhered to by older adults is therefore very important for the maintenance of high levels of well-being in later life. The pilot phase of the LIFE study [39] which targets physical function and mobility disability has reported very promising results. The investigation of the effectiveness and cost-effectiveness of similar approaches in the UK will provide important information in identifying ways to support high levels of well-being. It is possible that should programmes like this be seen as enjoyable, motivational and stimulating by older people they may also improve cognitive function and psychological well-being.

This study has some limitations. We used uniaxial accelerometers (Actigraph GT1M) to measure physical activity and sedentary time. Although accelerometry provides a valuable objective measure of activity, its validity for the assessment of sedentary time is not well-established and misclassifications could occur (e.g., between sitting and standing, and wear and non-wear time), therefore it may have limited capacity to detect relationships with well-being variables. In this study, MVPA includes all minutes of MVPA and not sustained 10 minute bouts. This may explain why participants’ MVPA is relatively high in comparison to national and international survey data. It is also possible that the OPAL study may have included people who are more active and functionally able than people over the age of 70 typically are due to the inclusion criteria of the study.

Technologies such as the activPAL [46], which records postural changes, may be more sensitive and depict a fuller picture of types of sedentary behaviours (i.e. sitting/lying, standing). We analysed the accelerometry data using what are currently accepted cut points, however these cut points are based on limited data and remain subject to debate. The cross-sectional nature of this observational study does not allow investigation of the direction of causality among the physical activity, sedentary time and well-being dimensions. Future studies, both longitudinal and experimental, will shed light onto the complex relationship between lifestyle behaviours and well-being. Finally, although our study recruited older people from twelve general practices to allow for representativeness, the majority of our participants were well-educated and in good health which might have attenuated relationships.

## 4. Conclusions

Physical activity, as measured by steps per day and amount of moderate to vigorous intensity activity, is independently moderately associated with perceived physical well-being in later life. However, its relationship with psychological well-being variables is weak. Sedentariness is a common behaviour amongst older adults but these findings suggest that it may not be related to subjective well-being. Programmes to increase physical activity are warranted for many reasons, but the improvement of perceptions of physical health and function may also be an important outcome, especially as physical self-perceptions are known to drive choice and persistence in further physical activity.
